# Oral Manifestations of Secondary Syphilis

**DOI:** 10.7759/cureus.103185

**Published:** 2026-02-07

**Authors:** Julie Rohban, Georgio Saad, Georges Khalil, Wassim Manhal

**Affiliations:** 1 Department of Oral Surgery, Faculty of Dental Medicine, Saint Joseph University of Beirut, Beirut, LBN; 2 Department of Microbiology, Faculty of Medicine, Saint Joseph University of Beirut, Beirut, LBN; 3 Department of Oral Medicine and Radiology, Faculty of Dental Medicine, Saint Joseph University of Beirut, Beirut, LBN

**Keywords:** infectious disease, oral manifestations, secondary syphilis, sexually transmitted infection, treponema pallidum

## Abstract

Syphilis is a chronic infectious disease caused by *Treponema pallidum*. It has resurfaced over the past years and remains a significant global public health problem. Its clinical presentation varies by stage, and oral lesions can be difficult to diagnose because they often resemble other pathologies. We report the case of a 28-year-old man presenting with indolent oral lesions, hoarseness, and dysphagia. A firm right-sided lymph node was also noted. Serological tests confirmed secondary syphilis. The patient received intramuscular benzathine penicillin G, leading to complete resolution of the lesions. Oral manifestations of secondary syphilis can present with diverse and nonspecific lesions, requiring careful clinical evaluation and appropriate serological testing for accurate diagnosis.

## Introduction

Syphilis is an ancient infectious disease that remains a major public health problem [1]. After the introduction of penicillin, the incidence of new cases declined considerably. However, the number of infections has been rising steadily since the early 2000s [2].

Syphilis is caused by the bacterium *Treponema pallidum *and may be either acquired or congenital [3,4]. Acquired syphilis is primarily transmitted through sexual contact or blood transfusion and progresses through several stages: primary, secondary, latent, and tertiary disease [5]. Congenital syphilis is transmitted from mother to child during pregnancy [3,4].

Among the clinical presentations of syphilis, oral signs may appear early in the course of the disease and across different stages of infection [6]. Oral manifestations are frequently observed during secondary syphilis, typically appearing weeks to months after the primary infection, and may be present in up to one-third of affected patients [6]. Syphilis progresses slowly, and its diagnosis is challenging because it manifests in various ways, earning the name “the Great Imitator” [1].

This case report highlights the diagnostic challenge posed by isolated oral lesions of secondary syphilis and underscores the importance of including syphilis in the differential diagnosis of atypical oral findings.

## Case presentation

A 28-year-old man presented to the Department of Oral Medicine and Radiology, Faculty of Dental Medicine, Saint Joseph University of Beirut, complaining of indolent oral lesions, hoarseness, and pain during swallowing that had persisted for the past month.

**Figure 1:**
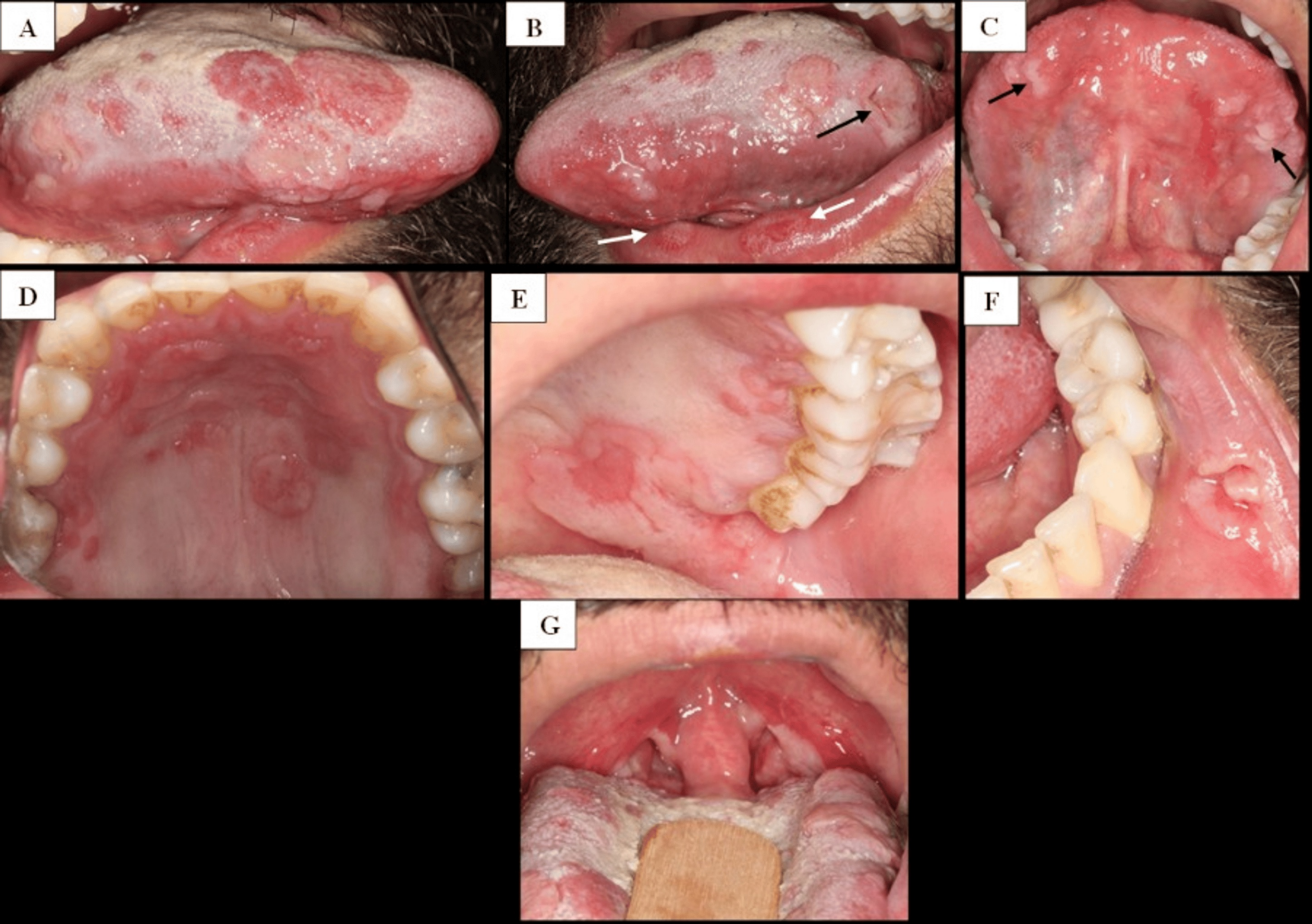
Intraoral lesions (pretreatment, day 0) (A) Right lateral view of the tongue showing multiple irregular erythematous patches (1-2 cm) with well-demarcated borders. (B) Left lateral view of the tongue showing multifocal atrophic and erythematous lesions (1-2 cm), including a round ulcerated plaque at the back (black arrow). Two red plaques are present on the vermilion border of the lower lip (white arrows). (C) Ventral surface of the tongue showing several white plaques (black arrows). (D) Occlusal view of the hard palate revealing multiple erythematous macules and patches, some with superficial ulceration, distributed bilaterally along the posterior and lateral palatal mucosa. (E) Left palatal retrotuberosity mucosa displaying an irregular erythematous patch with red eroded borders (~3 cm). (F) Inner aspect of the left lower lip showing an ulcerated lesion (~1 cm). (G) Anterior and posterior tonsillar pillars and the uvula showing erythema and coverage with white plaques.

Intraoral examination revealed multiple white and erythematous plaques, approximately 1-2 cm in diameter, involving the lateral, dorsal, and ventral surfaces of the tongue (Figure 1A-1C), the vermilion border of both the upper and lower lips (Figure 1B), and the hard palate (Figure 1D, 1E). Ulcerated lesions of similar size were observed on the left lateral border of the tongue and the inner aspect of the left lower lip (Figure 1B, 1F). In addition, the anterior and posterior tonsillar pillars, as well as the uvula, appeared erythematous and were covered with white plaques (Figure 1G).

Extraoral examination showed a firm, unilateral lymph node enlargement on the right side of the neck, measuring approximately 2-3 cm. The patient reported small, itchy papules on the genitals a few weeks before the onset of the oral lesions; however, no precise timing was available. He also reported a history of unprotected sexual activity during the anamnesis.

The clinical findings led to a presumptive diagnosis of secondary syphilis. Preliminary investigations included a complete blood count, *T. pallidum *hemagglutination assay (TPHA) and Venereal Disease Research Laboratory (VDRL) tests, HIV serology, and a sexually transmitted infections (STIs) panel.

**Figure 2:**
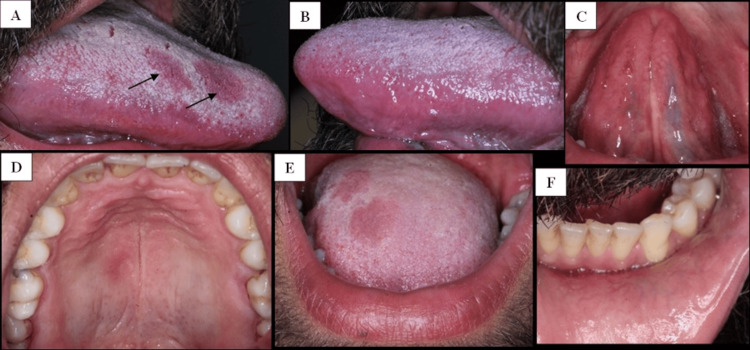
Intraoral findings following treatment of secondary syphilis (posttreatment, three weeks) (A) Right lateral view of the tongue showing resolution of previously observed erythematous and ulcerated plaques (black arrows indicate prior lesion sites). (B) Left lateral view of the tongue demonstrating complete healing of atrophic and erythematous lesions. (C) Ventral surface of the tongue showing absence of residual plaques. (D) Occlusal view of the hard palate revealing uniform mucosal coloration with the disappearance of previously observed erythematous macules and patches. (E) Vermilion border of the lower lip appearing normal. (F) Inner aspect of the left lower lip showing healing of the previously ulcerated lesion.

The TPHA was positive, with a value of 186 (>1, positive), and the VDRL test was also positive, confirming the diagnosis of secondary syphilis. A biopsy was not performed because of the characteristic clinical presentation and positive serology. The patient was treated with intramuscular benzathine penicillin G, 2.4 million units weekly for three weeks. The oral lesions resolved after treatment, and complete remission was achieved (Figure 2A-2F).

To improve clarity and reproducibility, a timeline summarizing symptom onset, investigations, treatment, and clinical response is presented in Table 1.

**Table 1:** Timeline of clinical presentation, investigations, treatment, and outcome TPHA, *Treponema pallidum *hemagglutination assay; VDRL, Venereal Disease Research Laboratory

Event	Date	Findings/results
Genital papules	A couple of weeks before the onset of oral lesions	Patient reported itchy genital papules
Oral lesions	Mid-February 2025 (per patient)	Asymptomatic oral lesions, hoarseness, and pain during swallowing
Consultation	March 18, 2025	Multiple erythematous and ulcerated plaques, hoarseness, and pain during swallowing
TPHA	March 24, 2025	186 (positive)
VDRL	March 24, 2025	Positive
Treatment start	March 28, 2025	Benzathine penicillin G 2.4 MU IM weekly × 3
Posttreatment	April 25, 2025	Complete resolution of lesions

## Discussion

Syphilis is a widespread STI affecting both teenagers and adults [7]. It is a chronic disease that, if left untreated, can progress through primary, secondary, and tertiary stages over the years [5,8,9]. Primary syphilis typically develops between three days and three months post-exposure, most commonly within two to three weeks after direct contact with an infectious lesion at the site of inoculation [9]. This stage is clinically characterized by the presence of a chancre. Chancres are usually single, firm, and painless ulcerations, although they can sometimes be painful or multiple [10]. They are highly contagious and develop at the site of inoculation, most commonly on the genital mucosa, but 4-12% are typically found on the lips, tongue, inside the cheeks, palate, gums, or tonsils [11]. Swollen lymph nodes, which may be painful or painless, can also be present. The lesions generally resolve on their own within three to eight weeks, and because they are often asymptomatic, only 30-40% of individuals are diagnosed during this early stage [10,11].

Unlike the single chancre of primary syphilis, secondary syphilis usually presents with multiple lesions, appearing two to 12 weeks later. Secondary syphilis is characterized by non-itchy skin rashes and mucosal lesions, sometimes accompanied by systemic symptoms such as fatigue, muscle aches, sore throat, fever, and headache [12]. Mucous patches are seen in around 30% of cases: white or pink lesions on the lips, tongue, cheeks, or palate. They may occasionally form “split papules” at the corners of the mouth [11,12]. If untreated, syphilis may progress to a latent stage and eventually to tertiary syphilis, even after many years, though this is uncommon [13]. Tertiary syphilis affects about 30% of patients and can damage multiple organs, including the nervous system in the form of neurosyphilis. Gummas, which can appear on the tongue or palate, are reported in tertiary syphilis [14].

Syphilis may present with nonspecific oral manifestations and can mimic various other pathologies. In our case, we observed multiple white and red plaques on the hard palate, the vermilion border of both upper and lower lips, and the lateral, dorsal, and ventral surfaces of the tongue. Ulcerated lesions were present on the left lateral border of the tongue and the inner aspect of the left lower lip. Furthermore, the anterior and posterior tonsillar pillars, as well as the uvula, were erythematous and covered with white plaques. Because these lesions can be present in other diseases, the differential diagnosis of syphilis may include oral manifestations of Crohn’s disease, erosive lichen planus, drug-related ulcers, traumatic ulcerations, aphthous ulcerations, geographic tongue, and squamous cell carcinoma [9]. Nevertheless, according to the literature, secondary syphilis can present with white or pink lesions on the lips, tongue, cheeks, or palate and may be accompanied by systemic symptoms such as fatigue and sore throat [6]. Our patient exhibited typical lesions with hoarseness and dysphagia. Based on the anamnesis, syphilis was suspected, and TPHA and VDRL tests were performed.

After clinical examination, histological analysis can be performed via incisional biopsy, along with serological tests to confirm the diagnosis. Among the most frequently used serological tests are nontreponemal tests, such as the commonly used VDRL, and treponemal tests, including TPHA [15]. In this case, both TPHA and VDRL tests were performed because TPHA detects antibodies specific to *T. pallidum *and confirms exposure to the organism, whereas VDRL detects nontreponemal antibodies associated with active infection and disease activity [15]. The combination of positive TPHA and VDRL results supported the diagnosis of secondary syphilis in this patient.

Regarding treatment, the World Health Organization and the Centers for Disease Control and Prevention (2021) recommend a single intramuscular dose of benzathine penicillin G (2.4 million units) for early syphilis [16]. For late latent syphilis or syphilis of unknown duration, the recommended regimen consists of benzathine penicillin G 2.4 million units administered intramuscularly once weekly for three consecutive weeks (total dose: 7.2 million units) [17]. In the present case, this extended regimen was selected because the exact duration of infection could not be reliably determined, ensuring adequate therapeutic coverage.

For patients with penicillin allergy, doxycycline is recommended as an alternative at a dose of 100 mg orally twice daily for 14 days in early syphilis [18].

## Conclusions

Oral lesions of secondary syphilis can mimic other oral pathologies, making diagnosis challenging. This case emphasizes the importance of a meticulous clinical examination and a thorough understanding of the disease’s clinical presentation. Early diagnosis and appropriate treatment result in complete resolution of lesions. Follow-up serology and partner notification are essential to ensure effective management and prevent further transmission.
